# Genome comparison reveals that *Halobacterium salinarum* 63‐R2 is the origin of the twin laboratory strains NRC‐1 and R1

**DOI:** 10.1002/mbo3.1365

**Published:** 2023-06-13

**Authors:** Friedhelm Pfeiffer, Mike Dyall‐Smith

**Affiliations:** ^1^ Computational Biology Group Max‐Planck‐Institute of Biochemistry Martinsried Germany; ^2^ Veterinary Biosciences, Melbourne Veterinary School, Faculty of Science University of Melbourne Parkville New South Wales Australia

**Keywords:** Archaea, comparative genomics, haloarchaea, halobacteria, mobilome, plasmid

## Abstract

The genome of *Halobacterium* strain 63‐R2 was recently reported and provides the opportunity to resolve long‐standing issues regarding the source of two widely used model strains of *Halobacterium salinarum*, NRC‐1 and R1. Strain 63‐R2 was isolated in 1934 from a salted buffalo hide (epithet “cutirubra”), along with another strain from a salted cow hide (91‐R6^T^, epithet “salinaria,” the type strain of *Hbt. salinarum*). Both strains belong to the same species according to genome‐based taxonomy analysis (TYGS), with chromosome sequences showing 99.64% identity over 1.85 Mb. The chromosome of strain 63‐R2 is 99.99% identical to the two laboratory strains NRC‐1 and R1, with only five indels, excluding the mobilome. The two reported plasmids of strain 63‐R2 share their architecture with plasmids of strain R1 (pHcu43/pHS4, 99.89% identity; pHcu235/pHS3, 100.0% identity). We detected and assembled additional plasmids using PacBio reads deposited at the SRA database, further corroborating that strain differences are minimal. One plasmid, pHcu190 (190,816 bp) corresponds to pHS1 (strain R1) but is even more similar in architecture to pNRC100 (strain NRC‐1). Another plasmid, pHcu229, assembled partially and completed in silico (229,124 bp), shares most of its architecture with pHS2 (strain R1). In deviating regions, it corresponds to pNRC200 (strain NRC‐1). Further architectural differences between the laboratory strain plasmids are not unique, but are present in strain 63‐R2, which contains characteristics from both of them. Based on these observations, it is proposed that the early twentieth‐century isolate 63‐R2 is the immediate ancestor of the twin laboratory strains NRC‐1 and R1.

## Graphical abstract

The complete genomes of four Halobacterium salinarum strains were compared in detail. Two strains (91‐R6^T^ and 63‐R2) were isolated in 1934 by Lochhead from cow and buffalo hides. From the results of these comparisons, we conclude that strain 63‐R2 is the immediate ancestor of the two, widely used laboratory strains NRC‐1 and R1.

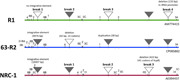

## 1 INTRODUCTION

The use of salt in the preservation of food (curing) and the tanning of leather are traditional processes dating back hundreds of years. In 1922, searching for the cause of “red discolorations” on salted codfish, which was seen as a threat to the Canadian fishery industry, Harrison and Kennedy isolated a red pigmented microorganism (Harrison & Kennedy, 1922), which they named *Pseudomonas salinaria*. This was the original isolate and designated the type strain of *Halobacterium salinarum* (according to the currently approved taxonomy). Later, this strain was lost.

In 1934, Lochhead investigated “red discolorations” (also called “red heat”) of salted hides, which were causing losses for Canadian leather manufacturers (Lochhead, 1934). During that study, he cultivated two more red‐pigmented isolates, one of which (91‐R6), obtained from a cow hide, was given the species epithet “salinaria” because of its high similarity with the organism isolated previously by Harrison and Kennedy. After the 1922 isolate was lost, 91‐R6^T^ was advanced as the type strain (neotype) of *Hbt. salinarum*. The other Lochhead isolate (63‐R2), obtained from a buffalo hide, was given the species epithet “cutirubra.” It was considered a distinct species by Lochhead, but this was later revised (Ventosa & Oren, 1996). The two Lochhead isolates from 1934 were deposited in the National Research Council (NRC) of Canada culture collection as NRC 34001 (63‐R2) and NRC 34002 (91‐R6). Although the NRC culture collection closed, the strains are preserved in several other culture collections (Grant et al., 2001), e.g ATCC 33170, DSM 669 (63‐R2) and ATCC 33171, DSM 3754 (91‐R6).

Taxonomically, strain 91‐R6^T^ is the type strain of *Hbt. salinarum*, and strain 63‐R2 is the type strain of *Halobacterium cutirubrum*, a name that has been validly published but is a younger heterotypic synonym of *Hbt. salinarum* according to the LPSN (list of prokaryotic names with standing in nomenclature) (Meier‐Kolthoff et al., 2022). The epithet *salinarum* has priority due to its earlier publication (1922, compared to 1934) according to the international code of nomenclature of prokaryotes (Parker et al., 2019). *Hbt. halobium* and *Hbt. cutirubrum* were designated species *incertae sedis* in 1996 (Ventosa & Oren, 1996), and since then organisms previously referred to by these names have been designated strains of *Hbt. salinarum*.

While the original Lochhead isolates (63‐R2 and 91‐R6) were only rarely used for experimental analyses, the twin pair of laboratory strains (NRC‐1, R1) was extensively studied. Both were assumed to be derived from *Hbt. salinarum* DSM 670, a strain obtained from the Stoeckenius lab which was referred to as *Hbt. halobium* (Stoeckenius & Kunau, 1968; Stoeckenius & Rowen, 1967). DSM 670 is thought to have come from NRC deposited strain NRC 34020 (Gruber et al., 2004). Attempts to retrieve their exact origin were not successful (Grant et al., 2001), but this can now be re‐evaluated using their genome sequences. DSM 671, strain R1, is the gas‐vesicle‐free mutant of DSM 670 (Stoeckenius & Kunau, 1968). It is from the purple membrane of strain R1 that Dieter Oesterhelt isolated bacteriorhodopsin (Oesterhelt & Stoeckenius, 1971), a light‐driven proton pump (Oesterhelt & Stoeckenius, 1973) which enables *Halobacterium* to grow by a second principle of photosynthesis (Oesterhelt & Krippahl, 1983).

High‐quality genome sequences for all four strains of *Hbt. salinarum* (91‐R6, 63‐R2, NRC‐1, R1) have now been determined, allowing detailed comparison and analysis. The genome sequence of *Hbt. salinarum* strain NRC‐1 was the first haloarchaeal genome sequence that became publicly available in 2000 (Ng et al., 2000), and also one of the first archaeal species sequenced. This has become the reference genome for halophilic archaea, with hundreds of literature citations. The complete genome sequence of strain R1 was published in 2008 (Pfeiffer et al., 2008), that of strain 91‐R6 in 2019 (Pfeiffer et al., 2019, 2020), and that of strain 63‐R2 in 2022 (DasSarma et al., 2022). Detailed interstrain comparisons revealed that the chromosomes of R1 and NRC‐1 are completely colinear and virtually identical (Pfeiffer et al., 2008). They are also highly similar (in silico DDH, 95%) to the type strain (91‐R6^T^) (Pfeiffer et al., 2020), confirming the taxonomic assignment of strain NRC‐1 to the species *Hbt. salinarum* (Gruber et al., 2004). The availability of the high‐quality genome sequence for strain 63‐R2 now allows the interstrain genome comparisons of all four strains.

A distinctive feature of *Hbt. salinarum* is a high rate of spontaneous mutation due to the movement of, and recombination between, mobile genetic elements (MGEs) (ISH elements, transposons, “the mobilome”), and this has been a focus of study from the 1980s onwards (DasSarma et al., 1983; Ng et al., 2000; Pfeifer & Blaseio, 1990; Pfeiffer et al., 2008, 2020). ISH elements are not only associated with insertional inactivation of genes but also genome inversions and other genome rearrangements (Ng et al., 1991; Pfeiffer et al., 2020). Most of the differences between the twin laboratory strains NRC‐1 and R1 could be attributed to this highly active mobilome (Pfeiffer et al., 2008).

In this study, the core genomes for all four strains were compared to assess the relationship between laboratory strains NRC‐1 and R1, and the original Lochhead strains 91‐R6, 63‐R2. In these comparisons, strain‐specific copies of MGEs were removed to reduce the background noise and enhance any evolutionary signals. The genome of strain 63‐R2 (NRC 34001) was found to be exceedingly similar to the laboratory twins NRC‐1 and R1, and the types of changes seen are consistent with strain 63‐R2 being the ancestral strain from which the two laboratory strains were derived. We believe that the origin of these laboratory strains has now been resolved.

## 2 MATERIALS AND METHODS

Detailed methods are provided in the supplementary material, deposited at Zenodo: https://doi.org/10.5281/zenodo.7780801. For convenience, summaries of these methods are given below.

### 2.1 Formatting the chromosomal sequences of strains 63‐R2, 91‐R6, NRC‐1, and R1 for comparative analysis

In‐house tagged versions of the genome sequences of strains 63‐R2, 91‐R6, R1, and NRC‐1 were generated in which all unique sequences between MGEs were identified, as well as each MGE and associated target sequence duplication (TSD).

After the removal of  comments, a “total” sequence was available for each strain. The concatenation of these sequences resulted in a “total” database for subsequent analyses, especially the determination of positions in the original genome sequences. In this “total” database file, line breaks around MGEs are preserved so that their visual identification is simple, especially when the MGE is enclosed by a TSD. A copy of that file served as the initial version of the “core” database, open for subsequent manual modification, most importantly the removal of strain‐specific copies of MGEs.

### 2.2 Chromosome comparison strategy and generation of core chromosomes devoid of strain‐specific MGEs for strains 63‐R2, NRC‐1 and R1

Preliminary genomic comparisons (BLASTn, MUMMer) had indicated that the genome sequence of strain 63‐R2 was much more closely related to those of the twin laboratory strains NRC‐1 and R1 than to that of strain 91‐R6^T^, and because of this, the initial analyses were restricted to these three strains. Applying an iterative comparison procedure, “core” chromosome sequences devoid of strain‐specific MGEs were generated. The build‐up of this “core” database is described in Supplementary Methods, deposited at Zenodo: https://doi.org/10.5281/zenodo.7780801. All eliminated MGEs, including their position in the original genome sequence, are documented in Table A1.

BLASTn analyses of the “core” sequences resulted in a complete set of HSPs (high‐scoring pairs) that correlated the complete “core” sequence of the chromosome from strain 63‐R2 against the core chromosomes from the twin laboratory strains NRC‐1 and R1.

HSP positions of the interstrain comparison are reported for the “core” database, but to allow easy correlation with biological features, all “core” database positions have been correlated with the corresponding positions in the original sequences of the “total” database (Supplementary Table S4, deposited at Zenodo: https://doi.org/10.5281/zenodo.7780801).

### 2.3 Comparison of the chromosome of strain 91‐R6 to the core chromosome from strain 63‐R2

The identification and elimination of MGEs that are specific for strain 91‐R6^T^ as compared to strains 63‐R2, NRC‐1, and R1 are described in Supplementary Methods: https://doi.org/10.5281/zenodo.7780801. Strain‐specific MGEs detected upon analysis of strain 91‐R6 either occur only in the chromosome of strain 91‐R6 (documented in Table A2), or are present in all three of the other strains (63‐R2, NRC‐1, and R1; documented in Table A1).

The core chromosomes of strains 91‐R6 and 63‐R2 were compared by BLASTn, leading to long HSPs, interrupted by unique sequences, which typically were short. Two long regions were encountered that are considered unique despite having a small number of short HSPs (see the Supplementary Methods for details).

### 2.4 Comparison of the reported plasmids pHcu235 and pHcu43 from strain 63‐R2 to plasmids pHS3 and pHS4, respectively, from strain R1

Preliminary comparisons (BLASTn) indicated that the sequence of plasmid pHcu235 from strain 63‐R2 is most closely related to plasmid pHS3 from strain R1, so these plasmids were compared in detail using the same procedure as described for chromosomal comparison (see above, Section 2.2). Plasmid pNRC200 from strain NRC‐1 showed a more patchy relationship and was not included in this analysis.

Preliminary comparisons (BLASTn) indicated that the unique, 2.3 kb sequence of plasmid pHS4 from strain R1 is closely related to a region on plasmid pHcu43 from strain 63‐R2. Thus, the sequences of these two plasmids were compared. A plasmid corresponding to pHS4 has not been reported for strain NRC‐1, and thus a plasmid from this strain was not included in the analysis.

The position of strain‐specific MGEs and their associated TSD which were removed upon generation of core plasmid sequences, are listed in Table A3 (pHcu235/pHS3) and Table A4 (pHcu43/pHS4). The final results of this analysis are the HSPs obtained with the “core” sequence of pHcu235 against the “core” sequence of plasmid pHS3 from strain R1 and the HSP obtained for the “core” sequences of pHcu43 against pHS4.

### 2.5 Validation that a plasmid corresponding to pHS4 from strain R1 is absent from strain NRC‐1

This is based on an analysis of Illumina sequence reads obtained upon resequencing of strain NRC‐1 (Kunka et al., 2020) (SRA:SRR9025102) and is described in Supplementary Methods: https://doi.org/10.5281/zenodo.7780801.

### 2.6 Assembly of strain 63‐R2 plasmids pHcu190 and pHcu229 from deposited PacBio read data

PacBio sequence reads for strain 63‐R2 have recently become available (DasSarma et al., 2022). Reads were downloaded from the SRA database (SRA:SRR16600243). Details of the assembly procedure are described in Supplementary Methods: https://doi.org/10.5281/zenodo.7780801.

When sequence duplications between contigs exceeded the length of even the longest PacBio reads, related plasmids were used to guide assembly at the junctions of these duplications. For plasmid pHcu190, plasmids pNRC100 and pHS1 were used as guide sequences, and a complete plasmid could be assembled. For plasmid pHcu229, plasmids pNRC200 and pHS2 were used as a guide. The assembly remained incomplete at both ends, due to a very long duplication between pHcu229 and pHcu190. No heterogeneities could be detected within this duplication, and thus the sequence of pHcu229 could be completed in silico by transferring the corresponding sequence from pHcu190. The sequences of pHcu190 and both versions of pHcu229 are deposited at Zenodo: https://doi.org/10.5281/zenodo.7288901.

### 2.7 Subassembly walking

Sequence duplications that exceed the length of PacBio reads cannot be resolved by regular assembly procedures. In this case, we applied a method that we refer to as “subassembly walking” which is described in Supplementary Methods: https://doi.org/10.5281/zenodo.7780801.

For subassembly walking attempts, we selected PacBio reads based on the following sequence features: (a) unique sequences from other strains which were not covered in the set of contigs from strain 63‐R2, (b) sets of PacBio reads selected according to a yet unexplored junction between a unique sequence and a duplication; this enabled the minimum length of the duplicated sequence which is connected to that junction to be determined, and (c) optional MGE's, where some reads contained the MGE‐free sequence version, while others exemplified the junction between the MGE and the adjacent unique sequence.

### 2.8 Assembly of strain 63‐R2 contigs contigDRAFT1 and contigDRAFT2 which represent the residuals of a plasmid that has integrated into the chromosome

Some sequences in strains R1 and NRC‐1 are strain‐specific and are not represented in the other strain (R1: 210 kb; NRC‐1; 15 kb) (Pfeiffer et al., 2008). Large parts of these strain‐specific sequences occur in strain 63‐R2. Nevertheless, some of the R1‐specific sequences were seemingly absent from this strain and it was attempted to validate their absence. Surprisingly, PacBio reads were identified which contain some of the R1‐specific sequences even though these occur neither in the chromosome nor in any of the assembled plasmids from strain 63‐R2 (case [a] in Section 2.7). Readsets were selected and assembled within Geneious (de novo assembly tool). Reads were also mapped to available contigs, including minor ones (e.g., short; low coverage; atypical connectivities of duplicated sequences). Emerging contigs were validated and/or extended by subassembly walking, resulting in contigDRAFT1 and contigDRAFT2.

### 2.9 Additional bioinformatics tools

As general tools, MUMMER v4 (Delcher et al., 2003) and the BLAST suite of programs v2.2 (Altschul et al., 1997; Johnson et al., 2008) were used for genome comparisons. All of the reported HSPs were obtained by BLASTn with default parameters except for three (−e 0.001; −F F; −C 0). Thus, low‐complexity filtering and composition‐based statistics were switched off. This slightly more stringent *e*‐value cutoff was chosen to reduce casual hits. The TYGS server (Meier‐Kolthoff & Göker, 2019) was used to query by whole genome comparison if strains represent novel species or belong to known species. Geneious Prime (version 2022.0.2) was used for read mapping and read assembly (Kearse et al., 2012).

## 3 RESULTS

### 3.1 Initial comparison of the genome of Lochhead strain 63‐R2 with that of other completely sequenced strains of *Hbt. salinarum*

Complete genome sequences consisting of both chromosomes and plasmids of the Lochhead strains 91‐R6 and 63‐R2, and the laboratory strains NRC‐1 and R1 were submitted to the TYGS server for taxonomy assignment based on comparison of complete genomes (Meier‐Kolthoff & Göker, 2019; Meier‐Kolthoff et al., 2022). This server accesses its database of genomes from known type strains, including the type strain of *Hbt. salinarum* (Lochhead strain 91‐R6) as well as to *Hbt. salinarum* DSM 669 (=NRC 34001 = Lochhead strain 63‐R2, previously “*Hbt. cutirubrum*”). The most relevant data for taxonomic analyses generated by the TYGS server (digital DNA‐DNA hybridization, formula d4, dDDH[d_4_]) are given in Table 1. All dDDH(d_4_) values were above 90% in comparison to the type strain 91‐R6^T^, confirming they are all strains of *Hbt. salinarum* because they exceed the 70% species delineation threshold (Meier‐Kolthoff & Göker, 2019). The twin laboratory strains NRC‐1 and R1 show an exceedingly close relationship to strain 63‐R2 (>99% dDDH[d_4_]) and are slightly less related (93%–94% dDDH[d_4_]) to strain 91‐R6.

**Table 1:** Genome‐based taxonomy analysis (TYGS) server results for the analyzed strains of *Halobacterium salinarum*.

Strain analyzed	Accessions	dDDH(d_4_) versus *Hbt. salinarum* type strain (91‐R6)	CI *d_4_*	dDDH(d_4_) versus “*Hbt. cutirubrum*” (63‐R2)	CI *d*_*4*_
63‐R2	CP085882–CP085884	94.6	92.9–95.9	99.8	99.6–99.9
NRC‐1	AE004437, AE004438, AF016485	92.1	90.0–93.8	99.3	98.9–99.5
R1	AM774415–AM774419	93.1	91.1–94.6	99.5	99.1–99.7
91‐R6	CP038631–CP038633	99.9	99.7–99.9	94.1	92.4–95.5

*Note*: The genomes of four strains of *Hbt. salinarum* (as defined by their GenBank accessions) were subjected to TYGS server analysis (accessed January, 2022). The dDDH(d4) values including their 95% confidence interval (CI) are reported for two reference genomes. At the time of analysis, “*Hbt. salinarum* type strain” (strain 91‐R6, DSM 3754) was represented in TYGS as a draft genome (WGS genome VRYN01), and “*Hbt. cutirubrum*” (strain 63‐R2, DSM 669) as draft genome (WGS genome JACHGX01).

At the time of analysis (January 2022) and within TYGS, strains 91‐R6 (NCBI WGS project VRYN01) and 63‐R2 (NCBI WGS project JACHGX01) were represented by draft genomes. Strain 63‐R2 is represented in the results from TYGS by the name under which it has been validly published (*Hbt. cutirubrum*) even though this name is flagged as a “younger heterotypic synonym” so that this strain is nowadays assigned to the species *Hbt. salinarum* (see above).

**Figure 1:**
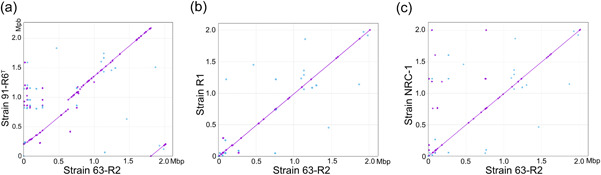
Dot plot (MUMMer) comparisons of *Halobacterium salinarum* strains 63‐R2, 91‐R6^T^, NRC‐1, and R1. The MUMMer tool was used to align and compare the published chromosomal sequence of *Hbt. salinarum* strain 63‐R2 with the chromosomes of strains 91‐R6^T^ (a), R1 (b), and NRC‐1 (c). Alignments were computed with the chromosome sequences as deposited in GenBank (see Table 1 for accessions).

This result was further corroborated by MUMMer comparisons of the chromosome sequences as deposited in GenBank (Figure 1). The MUMMer plot of the chromosomes from the Lochhead strains against each other (Figure 1a, strain 63‐R2 vs. strain 91‐R6) is dominated by a strong diagonal, but there is still one major and a few minor gaps (in addition to a breakpoint caused by selection of distinct start bases). The MUMMer plot of strain 63‐R2 against both of the laboratory strains (R1, NRC‐1, Figure 1b,c) consists of just a single completely contiguous diagonal starting at the left end of the chromosome and continuing right up to its right end.

### 3.2 Detailed comparison of the chromosomes from the most closely related strains, 63‐R2, NRC‐1, and R1

The chromosomes from the three extremely closely related strains 63‐R2, NRC‐1, and R1 were compared in detail using BLASTn. Due to the combination of extremely similar chromosome sequences and a highly active mobilome, mutations, which carry the evolutionary signal may be outnumbered by MGE mobilization events, which are of little relevance for unraveling the deeper evolutionary history of these strains. To avoid this problem, chromosome sequences devoid of strain‐specific MGEs were generated in silico (“core sequences”) and then used for comparison.

For transparency, all strain‐specific MGEs which were removed during this procedure are documented in Table A1. The final “core” chromosome of strain 63‐R2 was compared (BLASTn) to those of strains R1 and NRC‐1, and the resulting HSPs are listed in Tables 2a and 2b.

**Table 2a:** Comparison of the core chromosome sequences of strains 63‐R2 and R1.

Tag	Position (strain 63‐R2)	Position (strain R1)	Sequence identity (%)	Match bases/total bases	Gap characters	Comment
R_HSP1	1–170,773	1–170,773	99.99	170,772/170,773	0	‐
R_break1	170,774–180,249	8 bp overlap	‐	‐	‐	9476 bp insertion in strain 63‐R2
R_HSP2	180,250–589,134	170,766–579,649	99.99	408,881/408,885	1	‐
R_break2	13 bp overlap	579,650–579,678	‐	‐		14 codon deletion (pos 472–485) in transducer protein htrVI of strain 63‐R2 (LJ422_03260) compared to the orthologs from the laboratory strains (OE_2168R, VNG_0793G)
R_HSP3	589,122–1,095,986	579,679–1,086,558	99.99	506,862/506,880	17	‐
R_break3	Directly adjacent	38 bp overlap	‐	‐	‐	An insert in strain 63‐R2 in an intergenic region between divergently transcribed ORFs (OE_3125R and OE_3126F)
R_HSP4	1,095,987–1,861,458	1,086,521–1,852,001	99.99	765,470/765,481	9	‐
R_break4	1,861,459–1,861,562	29 bp overlap	‐	‐	‐	A 133 bp deletion in strain R1 in the rRNA promoter region
R_HSP5	1,861,563–1,997,337	1,851,973–1,987,747	100.00	135,775/135,775	0	‐

*Note*: The core chromosomes of strains 63‐R2 and R1 were compared using BLASTn (see also Figure 2). Core chromosomes are devoid of strain‐specific mobile genetic elements (MGEs) (see Table A1 for the coordinates of strain‐specific MGEs in the complete chromosome sequence). For all coordinates from the core chromosome, the corresponding coordinates from the complete chromosome are provided in Supplementary Table S4: https://doi.org/10.5281/zenodo.7780801. For HSPs (high‐scoring pairs, i.e., BLASTn alignment blocks), the start and end base is given. Also, raw counts (matching bases and total bases) as well as the number of gap characters, as returned by BLASTn, are listed. The % nucleotide sequence identity was recomputed (to provide two decimal point accuracy). High‐scoring pairs (HSP) are tagged R_HSP with a serial number (R to indicate comparison against strain R1). Regions that are not covered by HSPs are shown as breaks. The first and last base is given if there is an unaligned sequence. Otherwise, the term “directly adjacent” or, if applicable, the number of overlapping bases (bp; base pairs) is given. Breaks are tagged R_break with a serial number. For breaks, a comment briefly mentions the key aspect. In the core chromosome sequence of strain NRC‐1, the R1 specific break 1 is within N‐HSP1 (pos 170,773–180,248), the R1 specific break 4 is within N‐HSP4 (pos 1,861,061–1,861,164).

**Table 2b:** Comparison of the core chromosome sequences of strains 63‐R2 and NRC‐1.

Tag	Position (strain 63‐R2)	Position (strain NRC‐1)	Sequence identity (%)	Match bases/total bases	Gap characters	Comment
N_HSP1	1–589,134	1–589,132	99.99	589,128/589,134	2	‐
N_break1	13 bp overlap	589,133–589,161	‐	‐		See Table 2a, R_break2
N_HSP2	589,122–1,095,986	589,162–1,096,040	99.99	506,858/506,880	18	‐
N_break2	Directly adjacent	38 bp overlap	‐	‐	‐	See Table 2a, R_break3
N_HSP3	1,095,987–1,613,654	1,096,003–1,613,679	99.99	517,642/517,677	9	‐
N_break3	1,613,655–1,613,818	259 bp overlap	‐	‐	‐	164 extra bases in strain 63‐R2; a 423 bp deletion in strain NRC‐1 compared to strain R1 in the *hcpB* gene (VNG_2196G)
N_HSP4	1,613,819–1,997,337	1,613,421–1,996,939	99.99	383,517/383,519	0	‐

*Note*: The core chromosomes of strains 63‐R2 and NRC‐1 were compared by BLASTn (see also Figure 2). Core chromosomes are devoid of strain‐specific mobile genetic elements (MGEs) (see Table A1 for the coordinates of strain‐specific MGEs in the complete chromosome sequence). Tags start with N_ (to indicate comparison against strain NRC‐1). For further explanations of the Table layout, see the legend of Table 2a. In the core chromosome sequence of strain R1, the NRC‐1 specific break 3 is within R‐HSP4 (pos 1,604,198–1,604,361).

Due to the in silico removal of MGEs, the nucleotide positions listed in Tables 2a and 2b correspond to the core version and not to the original version of the genome sequence. The original nucleotide positions are provided in Supplementary Table S4, deposited at Zenodo: https://doi.org/10.5281/zenodo.7780801.

**Figure 2:**
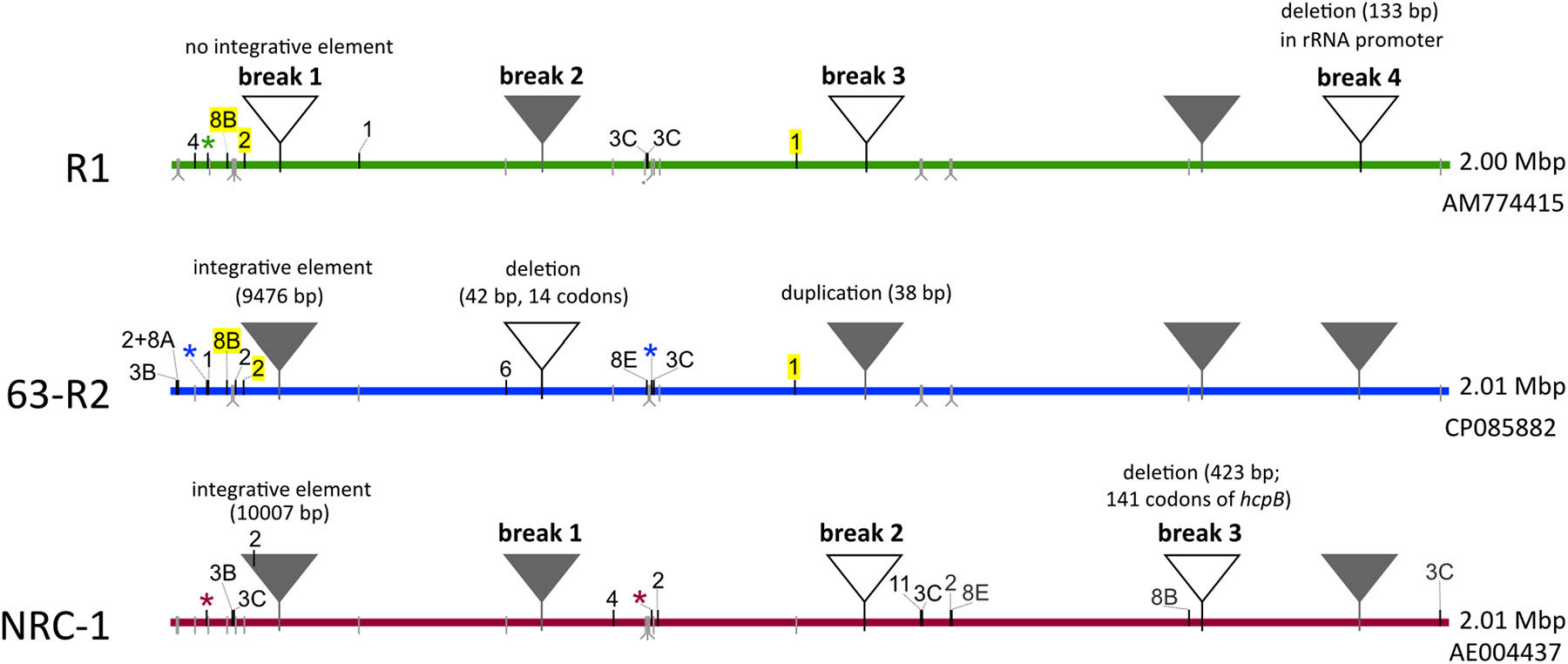
Breakpoints between the chromosomes from strains 63‐R2, NRC‐1, and R1. Colored solid lines represent the chromosomes of the three strains. The strain is indicated to the left, and the sequence length and accession are indicated to the right. Triangles indicate strain‐specific core sequences which result in an alignment break. Filled triangles indicate the presence and open triangles the absence of the strain‐specific sequence. The key characteristic of each break is indicated. For coordinates and details, see Tables 2a and 2b. Also shown are strain‐specific mobile genetic elements (MGEs) (black line extending above: present, MGE type indicated by a tag; gray line extending below: MGE absent; whiskers indicate the absence of multiple closely spaced MGEs). For MGEs that occur in two strains, tags are highlighted yellow. The three colored stars near the left end indicate distinct strain‐specific MGEs with distinct but closely spaced integration sites (serials 4–6 in Table A1). The two colored stars in the center indicate strain‐specific copies of the same MGE (ISH2) which are integrated at distinct but very closely spaced positions (872 bp apart, serials 21 and 22 in Table A1). The strain‐specific ISH2 in the integrative element of strain NRC‐1 is also indicated. Strain‐specific MGEs of category CB are not indicated because they do not differ among the represented strains.

All three chromosomes were found to be virtually identical, with only four (NRC‐1) and five (R1) HSPs, all showing 99.99% nucleotide sequence identity, needed to completely describe their relation to strain 63‐R2 (Tables 2a and 2b, Figure 2). There are only five events that lead to breakpoints, causing multiple HSPs (see Supplementary Text S1: https://doi.org/10.5281/zenodo.7780801). A duplication of only 38 bp is sufficient to be recorded as an event, which shows that the applied method is highly sensitive. Two breakpoints are novel and exemplify differences between strain 63‐R2 to both laboratory strains. The other three events are known from the comparison of the twin laboratory strains NRC‐1 and R1 (Pfeiffer et al., 2008).

### 3.3 Comparison of the core chromosomes from the two Lochhead strains 63‐R2 and 91‐R6

The chromosomal sequences of strains 63‐R2, 91‐R6, NRC‐1, and R1 were used for detailed analysis, but not all pairwise comparisons need to be performed because strain 91‐R6 had previously been compared to the twin laboratory strains NRC‐1 and R1 (Pfeiffer et al., 2020), using a distinct but related methodology (for result correlation see Table S2.10 and section S2.10 in Supplementary Text S2: https://doi.org/10.5281/zenodo.7780801). Also, due to the extreme similarity of strain 63‐R2 with strains NRC‐1 and R1, no additional relevant information can be gained by extending the comparison beyond the Lochhead strains. Thus, comparative data are only reported for strains 63‐R2 and 91‐R6; but in descriptions of the corresponding regions from strain 63‐R2, the near‐identical regions from the thoroughly analyzed laboratory strains are also referenced.

To facilitate the comparison of the chromosomes from the Lochhead strains 91‐R6 and 63‐R2, the start bases were adjusted as described in Supplementary Text S2: https://doi.org/10.5281/zenodo.7780801.

While only four or five HSPs from BLASTn comparisons are required to completely represent the relationship between Lochhead strain 63‐R2 and the twin laboratory strains NRC‐1 and R1, a much larger number of HSPs (38) is required to describe the relationship between the two Lochhead strains. These 38 HSPs sum up to 1,850,787 bp of which 1,844,079 are identical, giving an overall sequence similarity of 99.64%. The HSPs are separated by unique sequence breaks that are typically short (below 2 kb). Only a few of these are longer than 3 kb (11 of the unique regions). There are eight long breaks (3.2 kb to 9.4 kb), two very long pairs of unique sequences (47.0 to 78.2 kb), and one extremely long unique sequence (164.2 kb). Of the eight long breaks, six have features that are characteristic for proviruses (breaks 4, 12, 13 + 15, 25, 26). One long unique sequence codes for a type I restriction enzyme (break 2), and one long break is due to deletion of five genes, including *nrdAB* (break 34). Within two of the long breaks are a small number of homologous sequences which are short (typically less than 1 kb) and which show reduced sequence similarity (typically less than 90% nucleotide sequence identity). Details about each of them are provided in Supplementary Text S2: https://doi.org/10.5281/zenodo.7780801. Here, we only summarize key observations for the most relevant unique regions.

All breaks >10 kb are chromosomal replacements. The term replacement is used to refer to the result of a deletion‐coupled insertion (Dyall‐Smith et al., 2011). Strains 63‐R2, NRC‐1, and R1 have a well‐known AT‐rich island (61 kb) (Ng et al., 2000; Pfeifer & Betlach, 1985). This is replaced by a distinct AT‐rich island in strain 91‐R6 (47 kb) (Pfeiffer et al., 2020) (Table A7 and S1.1 in Supplementary Text S1; Table S2.1 in Supplementary Text S2, break 1: https://doi.org/10.5281/zenodo.7780801). A 78.2 kb sequence in strain 91‐R6 is replaced by a 44.1 kb sequence in strains 63‐R2, NRC‐1, and R1 (Supplementary Table S2.1, break 16). In both cases, several encoded proteins are closely related (up to 85% protein sequence identity) and even gene synteny is partially retained. However, there is little similarity at the nucleotide sequence level except for short patches of restricted sequence homology (short HSPs) (Supplementary Tables S2.3 and S2.7 in Supplementary Text S2: https://doi.org/10.5281/zenodo.7780801). Overrepresented among encoded proteins are glycosyltransferases and other enzymes acting on carbohydrates. These may be associated with the *N*‐glycosylation pathways or may be required for the production of extracellular polysaccharides (EPS).

The largest strain‐specific sequence is an insert in strain 91‐R6 (164.2 kb) which has characteristics of an integrated plasmid and which replaced 2.3 kb from strain 63‐R2, NRC‐1, and R1. A subregion, totaling 42.5 kb, shows a close but complex relationship to plasmid pHS3 (Pfeiffer et al., 2020).

The 164.2 kb unique sequence of strain 91‐R6 carries the genes *leuABCD* and *ilvBCDN* which code for enzymes of the branched‐chain amino acid biosynthesis pathway. Such enzymes are not encoded in the genomes of strains 63‐R2, NRC‐1, and R1. *Hbt. salinarum* is known to be auxotrophic for several amino acids. This might be related to the ample availability of proteins when grown on salted hides in a leather manufacturing environment.

### 3.4 Comparison of the reported plasmids from strain 63‐R2 and strain R1

**Figure 3:**
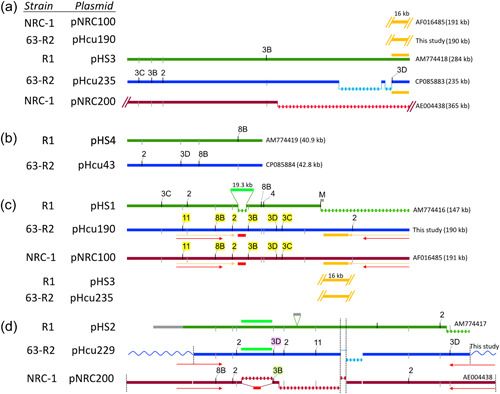
Correlation of the episomal plasmids of strain 63‐R2 with those from strains NRC‐1 and R1. Colored solid lines indicate colinear plasmid sequences (green: strain R1; blue: strain 63‐R2; red: strain NRC‐1). Strains and plasmids are indicated to the left and sequence length and accession are indicated to the right. The term “This study” is used for plasmids that have not been included in the original sequencing report and thus have not been deposited in GenBank. Loosely dashed lines indicate the absence of the corresponding sequence from the respective plasmid. All episomal plasmids are circular (not indicated). Plasmids and regions which are only partially displayed are indicated by a terminal slant line pair. Also shown are strain‐specific mobile genetic elements (MGEs) (black line extending above: present, MGE type indicated by a tag; gray line extending below: MGEs absent). For MGEs that occur in two strains, tags are highlighted yellow. There is additional, panel‐specific markup. (a) A 16 kb sequence that is duplicated between pHcu235 and pHcu190 from strain 63‐R2 is highlighted. The copy from pHcu235 is shared with pHS3 from strain R1 but is absent from pNRC200 from strain NRC‐1. For coordinates and details see Table 3. (b) For coordinates and details see Table 4. (c) A 16 kb sequence that is duplicated between pHcu235 and pHcu190 from strain 63‐R2 is highlighted. The copy from pHcu190 is shared with pNRC100 from strain NRC‐1 but absent from pHS1 from strain R1 due to a large deletion in that plasmid. The pHS1‐specific sequence “M” is indicated at the end of pHS1 (gray). A 4.5 kb sequence (indicated by a short light red line “below”) has been deleted in pHS1 and at the same position, a 19.3 kb sequence has been inserted (indicated by light green). This 19.3 kb sequence is also found in the same sequence context in pHS2/pHcu229 (indicated in [d] by a light green line “above”). The long inverted repeat is indicated by two pairs of arrows, the shorter (red, 32 kb) representing the extent of the duplication in pHcu229 and pNRC200, and the longer (orange, 40 kb) representing the extended duplication in pHcu190 and pNRC100. For coordinates and details see Tables 5a and 5b. (d) The sequence of pHcu229 could only be partially assembled due to extensive intra‐ and inter‐plasmid duplications. The vertical dashed lines at the termini indicate this incompleteness. The plasmid could be completed in silico (indicated by a wavy line) as it is most likely identical to the corresponding region of pHcu190, allowing a sequence transfer. A 19.3 kb sequence (indicated by a light green line “above”) has been deleted in pNRC200 and at the same position, a 4.5 kb sequence has been inserted (indicated by light red). This 4.5 kb sequence is also found in the same sequence context in pNRC100/pHcu190 (indicated in [c] by a light red line “below”). The inverted repeat is indicated by a pair of arrows (red, 32 kb) which correspond to the shorter arrows in (c). In pHcu229, the arrows traverse the termini of the assembled region and extend into the sequence added in silico. An ISH3D in pHcu229 (pink background) and an ISH3B in pNRC200 (light green background) are integrated into an identical sequence context (see Table A6). The vertical dashed line in the center indicates that the long deletion in pHcu229 partially overlaps with an independent long deletion in pNRC200. For coordinates and details see Tables 6a and 6b.

**Figure A1:**
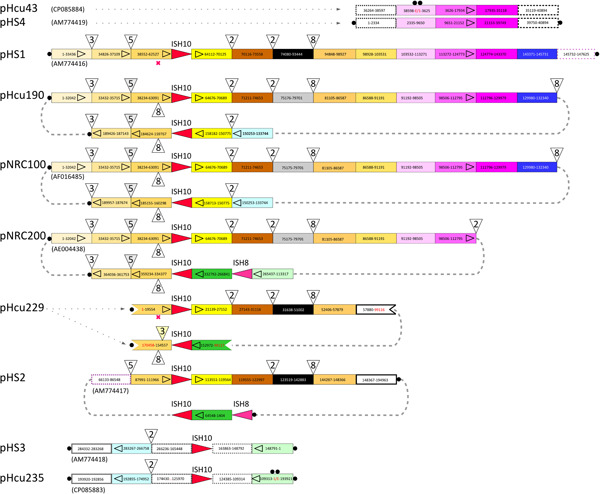
Comparative maps of the episomal plasmids of strains 63‐R2, NRC‐1, and R1. The episomal plasmids of the three strains show extensive intra‐ and interplasmid duplications. Plasmids having inverted duplications (pNRC100, pNRC200, pHcu190, and pHcu229) are drawn across two lines, with dashed connector lines indicating that the sequences are contiguous. Also, pHS2 is drawn in two lines to facilitate its comparison to closely related plasmids. For pHcu229, only the assembled part is drawn (indicated by a jagged end). The missing region is considered to be identical between pHcu190 and pHcu229 so that a complete sequence could be reconstructed in silico (not displayed). Coordinates for pHcu229 refer to the assembled version (170,458 bp) and not to the version completed in silico (229,124 bp). The start and end coordinates of each plasmid are marked by black dots. Shared regions (not drawn to scale) are indicated by identical coloring, except for regions in white boxes (see below for further details). Sequence directionality is indicated by box‐internal arrows. Each region is labeled by its coordinates (see below for further details). The regions in black (pHcu229, pHS1, pHS2, 19.3 kb) and gray (pHcu190, pNRC100, pNRC200, 4.5 kb) boxes are isopositioned alternative sequences. The region colored light blue is duplicated in strain 63‐R2 (pHcu190, pHcu235) but strains NRC‐1 and R1 have only a single copy with unrelated sequence context. The pHcu190 copy is absent from strain R1, the pHcu235 copy is absent from strain NRC‐1. The regions in white boxes are sequences unique to strain R1 as compared to strain NRC‐1 (seven regions). Five of these regions are shared between plasmids from strains R1 and 63‐R2 (drawn side by side and indicated by a common border pattern and color). Just two regions occur only in the episomal plasmids of strain R1 (within pHS2 and at the end of pHS1). The region at the end of pHS1 is present on a minor plasmid sequence that has been integrated into the chromosome (contigDRAFT1, not shown). Coordinates refer to the sequences in GenBank (accessions are provided toward the left end). If a region traverses the point of ring opening, this is indicated by E/1 in red font (in pHcu235, E is 235,323; in pHcu43, E is 42,817). Other coordinates in red font (in pHcu229) indicate that the region is incomplete at this end (also indicated by the jagged end). The incompleteness at the left end (start/end position) is due to incomplete assembly. The incompleteness at the right end is due to a 12 kb deletion. The three red dots in the coordinates of one region of pHcu235 refer to two closely spaced long deletions (44.2, 7.5 kb). Many region junctions are associated with transposons. These are indicated by triangles with numbers referring to the type of transposon. ISH5 transposons with a gray background are targeted by ISH11. The ISH10 transposons are drawn as an in‐line triangle (red), with directionality indicated. They are especially highlighted because the outer end of the inverted duplication in pNRC200 and pHcu229 (32 kb) is defined by this transposon. The inverted duplication of pNRC100 and pHcu190 extends further (by 8 kb, thus totalling 40 kb) and includes the region drawn in yellow. Also drawn as an in‐line triangle is an ISH8 transposon which marks the junction in pNRC200 between the region shared with pHS3 (regionN) and the region shared with pHS2 (regionT) (see Supplementary Table S3.4 in Supplementary Text S3: https://doi.org/10.5281/zenodo.7780801). This ISH8 is absent from pHcu229 due to a 12 kb deletion but is present on a minor plasmid sequence that has been integrated into the chromosome (contigDRAFT2, not shown). Additionally, two MGEs are drawn even though they are internal to a region. These are an ISH3 (highlighted yellow) which occurs exclusively in the reverse copy of the inverted duplication in pHcu229. An adjacent ISH8 is also indicated because it is present in the reverse copy of the inverted duplication of pHcu229 but absent from the forward copy (indicated by a small cross in red). This ISH8 is absent from the single copy of this region in plasmid pHS1 from strain R1 but present in all other copies of all other plasmids from strains 63‐R2 and NRC‐1.

Two plasmids have been reported for strain 63‐R2: pHcu235 and pHcu43 (DasSarma et al., 2022). An initial analysis revealed that these are very closely related to plasmids pHS3 and pHS4 from strain R1. The correlation between the plasmids from strain 63‐R2 and their most closely related counterparts from strains R1 and NRC‐1 is illustrated in Figure 3a,b and Figure A1.

A detailed description of the similarities and differences of these plasmids is available in Supplementary Text S3: https://doi.org/10.5281/zenodo.7780801.

Previous experience has shown that highly similar chromosomes can well be associated with largely unrelated plasmids (*Haloquadratum walsbyi* strains C23 and HBSQ001 [Dyall‐Smith et al., 2011]; *Hbt. salinarum* strain 91‐R6 compared to the laboratory strains NRC‐1 and R1 [Pfeiffer et al., 2020]). The fact that the plasmid sequences of strains 63‐R2 and R1 show 100% sequence identity over 231.1 kb (pHcu235 vs. pHS3, see Table 3) and 99.89% sequence identity over 39.5 kb (pHcu43 vs. pHS4, see Table 4) indicates that these strains are more closely related than is reasonable to assume for independent isolates. We thus consider it likely that strain R1 is a direct descendent from the cultured Lochhead strain.

**Table 3:** Comparison of the core sequences of plasmids pHcu235 from strain 63‐R2 and pHS3 from strain R1.

Tag	Position (pHcu235)	Position (pHS3)	Sequence identity (%)	Match bases/total bases	Gap characters	Comment
pHcu235_HSP1	1–210,501	1–210,501	100.00	210,501/210,501	0	
pHcu235_ break1	549 bp overlap	210,502–254,717				44,216 bp deletion in pHcu235
pHcu235_HSP2	209,953–212,491	254,718–257,256	100.00	2539/2539	0	
pHcu235_ break2	Directly adjacent	257,257–264,819				7563 bp deletion in pHcu235
pHcu235_HSP3	212,492–230,601	264,820–282,929	100.00	18,110/18,110	0	

*Note*: The core sequences of plasmids pHcu235 and pHS3 were compared using BLASTn (see also Figure 3a). Core plasmid sequences are devoid of strain‐specific mobile genetic elements (MGEs) (see Table A3 for the coordinates of strain‐specific MGEs in the complete plasmid). For further explanations of the table layout, see the legend of Table 2a.

**Table 4:** Comparison of the core sequences of plasmids pHcu43 from strain 63‐R2 and pHS4 from strain R1.

Tag	Position (pHcu43)	Position (pHS4)	Sequence identity (%)	Match bases/total bases	Gap characters
pHcu43_HSP1	1–39,479	1–39,481	99.89	39,440/39,483	6

*Note*: The core sequences of plasmids pHcu43 and pHS4 were compared using BLASTn (see also Figure 3b). Core plasmid sequences are devoid of strain‐specific mobile genetic elements (MGEs) (see Table A4 for the coordinates of strain‐specific MGEs in the complete plasmid). For further explanations of the table layout, see the legend of Table 2a.

### 3.5 Assembly of additional plasmids of strain 63‐R2 from the PacBio reads deposited at the SRA database

Because the two plasmids reported for strain 63‐R2 (DasSarma et al., 2022) proved to be exceedingly similar to two of the four plasmids reported for strain R1 (Pfeiffer et al., 2008), the presence of additional plasmids in strain 63‐R2 was investigated. Among the contigs generated by canu from the PacBio reads deposited at the SRA database (Section 2.6 above and Supplementary Methods) were the regenerated plasmids pHcu235 and pHcu43 previously reported by DasSarma et al. (2022). Among the other contigs were plasmid sequences, from which one plasmid could be finalized (pHcu190), and another partially assembled (pHcu229). Long repeats constrained the assembly of the latter plasmid to 170 kb but it could be expanded in silico to its predicted full length of 229 kb. The correlation between the novel plasmids from strain 63‐R2 and their most closely related counterparts from strains R1 and NRC‐1 is illustrated in Figure 3c,d. The subregions of the various episomal plasmids from strains 63‐R2, NRC‐1, and R1 are depicted in Figure A1.

Two additional contigs were obtained which reflect the integration of a plasmid into the chromosome. They share a large region in common but proved resistant to finalization despite detailed scrutiny. They are reported as contigDRAFT1 and contigDRAFT2.

#### 3.5.1 Assembly of plasmid pHcu190 from strain 63‐R2 and its comparison to pNRC100 from strain NRC‐1 and pHS1 from strain R1

Plasmid pHcu190 was assembled as a complete, circularized plasmid. It is closely related to plasmid pHS1 from strain R1 (Figure 3c), with the main difference being the replacement of a short region (1.9 kb, pHS1) by a much longer region (58.4 kb, pHcu190). Despite these deviations, the extreme similarity between pHcu190 and R1 (99.98% sequence identity over 120.6 kb) supports our hypothesis that strain R1 is a direct descendent of the cultivated Lochhead strain. Even more remarkable is the extreme similarity to plasmid pNRC100 from strain NRC‐1. After the removal of strain‐specific MGEs (see Table A5), pHcu190 and pNRC100 could be fully described by a single 183.6 kb HSP with 99.99% nucleotide sequence identity (Table 5a, Figure 3c). Notably, pHcu190 also carries the long inverted duplication which is known from pNRC100 (Ng et al., 1998) but is absent from pHS1. The remarkable similarity between pHcu190 and pNRC100 makes it likely that strain NRC‐1 is also a direct descendent from the cultured Lochhead strain 63‐R2. In summary, the plasmids from strain 63‐R2 display “hybrid characteristics” compared to the plasmids of strains NRC‐1 and R1, and unify seemingly inconsistent characteristics of the lab twin plasmids. This is further corroborated by the 16 kb sequence that matches between the unrelated plasmids pNRC100 and pHS3, being seemingly “shifted.” This 16 kb sequence is duplicated in strain 63‐R2, occurring in pHcu190, the equivalent of pNRC100, and in pHcu235, the equivalent of pHS3. The most parsimonious interpretation is that each of the laboratory strains has inherited plasmid precursors with both copies and then deleted one copy upon laboratory cultivation.

**Table 5a:** Comparison of the core sequences of plasmids pHcu190 from strain 63‐R2 and pNRC100 from strain NRC‐1.

Tag	Position (pHcu190)	Position (pNRC100)	Sequence identity (%)	Match bases/total bases	Gap characters
N_pHcu190_HSP1	1–183,605	1–183,604	99.99	183,597/183,605	1

*Note*: The core sequences of plasmids pHcu190 and pNRC100 were compared using BLASTn (see also Figure 3c). Core plasmid sequences are devoid of strain‐specific mobile genetic elements (MGEs) (see Table A5 for the coordinates of strain‐specific MGEs in the complete plasmid). For further explanations of the table layout, see the legend of Table 2a.

Two HSPs and two intervening unrelated sequences are required and sufficient to fully describe the relationship between pHcu190 and the R1 plasmid pHS1. The matching regions are 72.1 kb with 99.99% nucleotide sequence identity and 48.4 kb with 99.95% nucleotide sequence identity (Table 5b, Figure 3c). The first unique region is 4.5 kb in pHcu190 and 19.3 kb in pHS1. These strain‐specific regions were described previously in the comparison of pNRC100 and pHS1 (Pfeiffer et al., 2008) and are illustrated in Figure 3c and Figure A1. The 19.3 kb sequence in pHS1 is absent from the plasmids of strain NRC‐1, but present in another plasmid from strain 63‐R2 (pHcu229, see below). The other unique region is 58.4 kb in pHcu190 and covers the long (40 kb) inverted duplication and a 16 kb sequence which also occurs in pHcu235 (Figure 3c). This is replaced by a 1.9 kb region, carrying a copy of ISH2, in pHS1. Although the 1.9 kb sequence is absent from the plasmids of strain NRC‐1, and from the assembled plasmids of strain 63‐R2 (pHcu235, pHcu43, pHcu190, and pHcu229), it was detected in the PacBio reads of strain 63‐R2 as a 1.3 kb sequence without ISH2 (see below, contigDRAFT1). With respect to the inverted duplication, it may be speculated that it was present when strain 63‐R2 was cultivated by Lochhead, was retained in strain NRC‐1, and was initially also present in the lineage to strain R1 but was subsequently lost upon laboratory cultivation.

**Table 5b:** Comparison of the core sequences of plasmids pHcu190 from strain 63‐R2 and pHS1 from strain R1.

Tag	Position (pHcu190)	Position (pHS1)	Sequence identity (%)	Match bases/total bases	Gap characters	Comment
R_pHcu190_HSP1	1–72,155	1–72,155	100.00	72,155/72,155	0	
R_pHcu190_break1	72,156–76,681	72,156–91,519	‐			4526 bp region specific to pHcu190/pNRC100; 19,364 bp region specific to pHS1
R_pHcu190_HSP2	76,682–125,134	91,520–139,972	99.95	48,433/48,453	0	
R_pHcu190_break2	125,135–183,605	139,973–141,861	‐			1889 bp terminal region specific for pHS1; 58,471 bp region specific for pHcu190/pNRC100, which includes a 16 kb sequence and the long (40 kb) inverted duplication

*Note*: The core sequences of plasmids pHcu190 and pHS1 were compared using BLASTn (see also Figure 3c). Core plasmid sequences are devoid of strain‐specific mobile genetic elements (MGEs) (see Table A5 for the coordinates of strain‐specific MGEs in the complete plasmid). For further explanations of the table layout, see the legend of Table 2a.

#### 3.5.2 Detection and assembly of plasmid pHcu229 from strain 63‐R2, which is related to plasmid pHS2 from strain R1 and plasmid pNRC200 from strain NRC‐1

With the newly assembled pHcu190, three of the four plasmids from strain R1 (pHS1, pHS3, and pHS4) have an equivalent in strain 63‐R2. The PacBio reads from genome sequencing of strain 63‐R2 were successfully scrutinized for matches to unique regions from R1 plasmid pHS2. Using a supervised approach within Geneious, pHS2 as a reference, and subassembly walking (see methods and Supplementary Methods), a contig was assembled that is longer than 170 kb. Despite considerable efforts, it was not possible to further extend this contig which runs at both termini into the long inverted duplication known from pHcu190, pNRC100, and pNRC200 (Figure A1). Extensive attempts to detect reads which indicate additional heterogeneities between this plasmid and pHcu190 were not successful. Thus we assume that this plasmid is identical to pHcu190 in the overlapping region and completed its sequence in silico by inserting the corresponding region from pHcu190. The complete sequence was 229,124 bp and the plasmid was accordingly designated pHcu229.

After the removal of strain‐specific MGEs (see Table A6), three HSPs are required and sufficient to describe the relation of plasmid pHcu229 from strain 63‐R2 and pHS2 from strain R1 (Table 6a, Figure 3d). Also, three HSPs are required and sufficient to describe the relation of pHcu229 and pNRC200 from strain NRC‐1 (Table 6b, Figure 3d). However, the HSPs which describe the relation to pHS2 and those which describe the relation to pNRC200 are categorically different. pHcu229 is very similar to pHS2, in contrast to pNRC200, because just two simple events (one insertion, one deletion) are sufficient to describe all the observed differences. Overall, pHcu229 and pHS2 show more than 99.99% sequence identity over 151.0 kb, further supporting the hypothesis that strain R1 is a direct descendent of strain 63‐R2. Further comparison details are reported in Supplementary File S3: https://doi.org/10.5281/zenodo.7780801.

**Table 6a:** Comparison of the core sequences of plasmids pHcu229 from strain 63‐R2 and pHS2 from strain R1.

Tag	Position (pHcu229)	Position (pHS2)	Sequence identity (%)	Match bases/total bases	Gap characters	Comment
Incompleteness	‐	65,602–91,881	‐			No upstream match because pHcu229 has only been partially assembled (in silico extension not considered)
R_pHcu229_HSP1	1–67,160	91,882–159,041	99.99	67,159/67,160	0	
R_pHcu229_break1	11 bp overlap	159,042–163,202	‐			A 4161 bp transposon cassette in pHS2
R_pHcu229_HSP2	67,150–95,583	163,203–191,636	100.00	28,434/28,434	0	
R_pHcu229_break2	Directly adjacent	191,637–E/1‐10,161	‐			E:194432
R_pHcu229_HSP3	95,584–151,023	10,162–65,601	100.00	55,440/55,440	0	
R_pHcu229_break3	151,024–165,531	‐	‐			Present in pHcu229 and pNRC200 but absent from pHS2

*Note*: The core plasmid sequences of pHcu229 (restricted to its assembled region) and pHS2 were compared using BLASTn (see also Figure 3d). Core plasmid sequences are devoid of strain‐specific mobile genetic elements (MGEs) (see Table A6 for the coordinates of strain‐specific MGEs in the complete plasmid). For further explanations of the table layout, see the legend of Table 2a.

**Table 6b:** Comparison of the core sequences of plasmids pHcu229 from strain 63‐R2 and pNRC200 from strain NRC‐1.

Tag	Position (pHcu229)	Position (pNRC200)	Sequence identity (%)	Match bases/total bases	Gap characters	Comment
Incompleteness		1–42,124				No upstream match because pHcu229 has only been partially assembled (in silico extension not considered)
N_pHcu229_HSP1	1–31,107	42,125–73,231	100.00	31,107/31,107	0	
N_pHcu229_break1	31,108–50,471	73,232–77,757				19,364 bp region in pHcu229; 4526 bp region in pNRC200
N_pHcu229_HSP2	50,472–55,954	77,758–83,240	100.00	5483/5483	0	
N_pHcu229_break2	55,955–95,583	83,241–275,058				39,629 bp in pHcu229;191,818 bp in pNRC200
N_pHcu229_HSP3	95,584–165,531	275,059–348,884	99.99	61,062/61,063	0	
Incompleteness	‐	348,885–361,547				No downstream match because pHcu229 has only been partially assembled (in silico extension not considered)

*Note*: The core plasmid sequences of pHcu229 (restricted to its assembled region) and pNRC200 were compared using BLASTn (see also Figure 3d). Core plasmid sequences are devoid of strain‐specific mobile genetic elements (MGEs) (see Table A6 for the coordinates of strain‐specific MGEs in the complete plasmid). For further explanations of the table layout, see the legend of Table 2a.

#### 3.5.3 Integration of a plasmid into the chromosome, various sequence heterogeneities, and long contigs (contigDRAFT1 and contigDRAFT2)

Each plasmid from strain R1 has its equivalent in strain 63‐R2, despite a few notable differences. Thus, many sequences, which are R1 specific when compared to strain NRC‐1 (210 kb total) are common when compared to strain 63‐R2. To confirm that the residual R1‐specific sequences were truly absent from strain 63‐R2, the complete set of PacBio reads was searched for these sequences.

Unexpectedly, two R1 specific sequence regions were represented in the 63‐R2 PacBio reads even though they occurred neither in the chromosome of strain 63‐R2 nor in any of the four plasmids of this strain (pHcu235, pHcu43, pHcu190, and pHcu229). Contigs were iteratively extended by subassembly walking resulting in two contigs (contigDRAFT1, 51,618 bp; contigDRAFT2, 242,404 bp) which proved resistant to further extension. Both represent plasmid sequences that have integrated into the chromosome at position 1.190 Mb. Extensive duplications were encountered. ContigDRAFT1 and contigDRAFT2 have a common sequence of 24.2 kb and they duplicate regions of the plasmids from strain 63‐R2.

The duplication between pHcu190 and contigDRAFT1 in strain 63‐R2 is caused by the heterogenous PacBio reads and thus must reflect strain‐internal variability. It is undecided if ATCC 33170 (63‐R2) contains a mixed cell population or if the heterogenous sequences occur within the same cell. However, it is remarkable that both sequence versions known from the twin laboratory strains NRC‐1 and R1 occur in strain 63‐R2. Given that the previously designated “R1 specific” sequences were subsequently detected in contigDRAFT1 and contigDRAFT2 of strain 63‐R2, they must have been ancestral to strains 63‐R2, NRC‐1, and R1. Consequently, the insertion of a 1.9 kb sequence in pHS1 at the expense of a deletion of 58.4 kb was not an event that occurred in strain R1 but had occurred earlier in the lineage leading to this strain (see Supplementary Text S3, section S3.8). The most parsimonious interpretation is that these three strains originate from a single cultivation event, the isolation of strain 63‐R2 by Lochhead in 1934. Later, at unknown time points, samples were taken, probably independently, and further cultivated as either strain NRC‐1 or as the immediate parent (DSM 670) of the spontaneous gas‐vesicle‐free mutant strain R1 (DSM 671). At the same time, the originally cultivated cells have been further expanded as strain 63‐R2, and additional events likely altered the genome before the cells were deposited as ATCC 33170 and subsequently sequenced. This would explain the two novel breakpoints in strain 63‐R2, while strains R1 and NRC‐1 are identical in these regions. Details about the contigDRAFT1, contigDRAFT2, and the various duplicated regions in strains 63‐R2, NRC‐1, and R1 are provided in Supplementary File S3: https://doi.org/10.5281/zenodo.7780801.

## 4 DISCUSSION

The origin of the widely used laboratory strains of *Hbt. salinarum*, R1, and NRC‐1 was previously unclear, although genome comparisons had shown their chromosomes and plasmids were extremely similar, confirming that they both came from the same parental strain (Pfeiffer et al., 2008). Our previous genomic comparison of strains R1 and NRC‐1 with Lochhead strain 91‐R6^T^ (type strain of the species) excluded strain 91‐R6^T^ as being the parent of the two laboratory strains (Pfeiffer et al., 2020). The current study, analyzing a genome sequence that has recently been published (DasSarma et al., 2022), now establishes that parent as being strain 63‐R2, originally isolated from microbially spoiled buffalo hide by Lochhead and deposited in the NRC culture collection as NRC 34001 (Lochhead, 1934). Much of the previous confusion was caused by a combination of factors, including the difficulties in taxonomy before the sequencing era, changes in nomenclature, inadequate strain description in early research publications, and the closure of the Canadian culture collection without archiving strain documents.

The activity of the mobilome is known to dominate strain differences, especially for chromosomes, while nonmobilome‐related differences are extremely rare. To focus on nonmobilome differences, all strain‐specific MGEs were first removed in a clearly documented procedure that maximized transparency. The core genomes were then compared in detail.

Earlier insights gathered from changes observed in strains of *Hqr. walsbyi* (Dyall‐Smith et al., 2011) had unraveled two processes leading to gross differences between very closely related strains: deletion‐coupled insertion and repeat‐mediated deletion. Multiple examples of both processes were encountered in the current study.

A deletion‐coupled insertion results in a replacement so that unrelated sequences occur in an identical genomic context. Several differences between the two Lochhead strains can be attributed to deletion‐coupled insertion. One case is the 61 kb AT‐rich island of strain 63‐R2 which was already known from strains NRC‐1 and R1 (Ng et al., 2000; Pfeifer & Betlach, 1985) and which is replaced by an isopositioned 47 kb AT‐rich sequence in strain 91‐R6 (Pfeiffer et al., 2020). Another case is a 2.3 kb sequence in strain 63‐R2 which is replaced by a 164 kb plasmid‐like sequence of strain 91‐R6. Such very asymmetric cases of deletion‐coupled insertion have also been encountered in *Haloquadratum* (Dyall‐Smith et al., 2011). Also, a 44 kb sequence in strain 63‐R2 was replaced by a 78 kb sequence in strain 91‐R6. The latter replacement is remarkable because the 78 kb sequence in strain 91‐R6 contains a cluster of genes coding for enzymes involved in branched‐chain amino acid biosynthesis. In contrast, strain 63‐R2 and with it the twin laboratory strains NRC‐1 and R1 lack this biosynthetic pathway. Such a loss might only be possible in an environment that ensures a continuous supply of a protein‐rich diet, as is the case with spoilage of hides and leather during processing.

The chromosomes of strains 63‐R2 and NRC‐1 contain the same integrative element (ca 10 kb) which is absent from strain R1. This element has integrated into the *pilB2* gene and is associated with an 8 bp terminal duplication as a direct repeat. Its removal in R1 could either have been by precise self‐excision of the element but more likely by repeat‐mediated deletion, mediated by the 8 bp duplication.

The evolutionary signals conveyed by the plasmids of strains 63‐R2, NRC‐1, and R1 were also intriguing. While they are extremely well conserved in sequence, the architecture of the plasmids carried by these strains varied greatly. In a previous report (Pfeiffer et al., 2008), this was mistakenly taken as evidence of plasmid misassembly. However, both plasmids are well supported by experimental data, with evidence for the plasmids of strain NRC‐1 being even stronger (detailed restriction analyses) (Bobovnikova et al., 1994; Kennedy, 2005; Ng & DasSarma, 1991; Ng & Kothakota, & DasSarma, 1991; Ng et al., 1993, 1998, 2000, 2008) than that for the plasmids from strain R1 (cosmid end sequencing) (Pfeiffer et al., 2008).

Strain 63‐R2 carries four plasmids and, additionally, a clear residual signature of two versions of an integrated plasmid, the latter only with low sequence coverage. Only two of these plasmids have been reported by the authors who described the genome sequence of strain 63‐R2 (DasSarma et al., 2022): pHcu235, which is near‐identical to plasmid pHS3 from strain R1 and pHcu43, which is near‐identical to plasmid pHS4 from strain R1. An effort to validate the absence of equivalents to plasmids pHS1 and pHS2 resulted in the surprising detection of PacBio reads for corresponding plasmids in strain 63‐R2. As another unexpected discovery in these data, one plasmid, pHcu190, proved near‐identical to plasmid pNRC100 from strain NRC‐1 rather than to plasmid pHS1 from strain R1. The other plasmid, pHcu229, which could only be partially assembled due to extremely long perfect duplications, showed hybrid similarities. One part proved to be near‐identical to pHS2 from strain R1, while another part proved to be near‐identical to plasmid pNRC200 from strain NRC‐1. The extreme similarity of the plasmid sequences, despite variations in their architecture, calls for a direct genealogical descent rather than representing independent isolates. The Lochhead strain 63‐R2 is a well‐documented original isolate and most likely the immediate ancestor of the laboratory strains NRC‐1 and R1.

The major architectural difference between the plasmid pair pHcu190/pNRC100 and pHS1 is the presence of a 40 kb inverted duplication in the former and the absence of this in the latter. Being present in two strains, it can be assumed that the plasmid version including the inverted duplication is ancestral. The conversion of that presumed ancestral plasmid to pHS1 might well have been caused by deletion‐coupled insertion. The inserted sequence is 1.9 kb in length, and the deleted sequence is 58.4 kb in length and covers the inverted copy of the 40 kb deletion plus a 16 kb sequence, which is duplicated only in strain 63‐R2 (on pHcu235 and pHcu190) while only one copy is found in strain R1 on the pHcu235‐related plasmid pHS3, and only one copy is found in strain NRC‐1 on the pHcu190‐related plasmid pNRC100.

In a surprising discovery, while attempting to confirm the absence of the pHS1‐specific 1.9 kb sequence from strain 63‐R2, PacBio reads were found that carry this sequence (lacking an ISH2 element and thus being a 1.3 kb sequence). Expansion of that sequence uncovered remnants of a plasmid that is found integrated into the chromosome and which occurs in two variants. Thus, both architectures, that of plasmids pHcu190/pNRC100 and that of plasmid pHS1 must already have occurred in the common ancestor of strains 63‐R2, NRC‐1, and R1. Similarly, one variant of the integrated plasmid contains a junction that is specific to pNRC200 and joins sequences from pHS3 and pHS2 (and thus also from pHcu235 and pHcu229). Again, both architectures, that of plasmids pHS3 and pHS2 and that of pNRC200 must already have occurred in the common ancestor, which probably is the original isolate 63‐R2.

**Figure 4:**
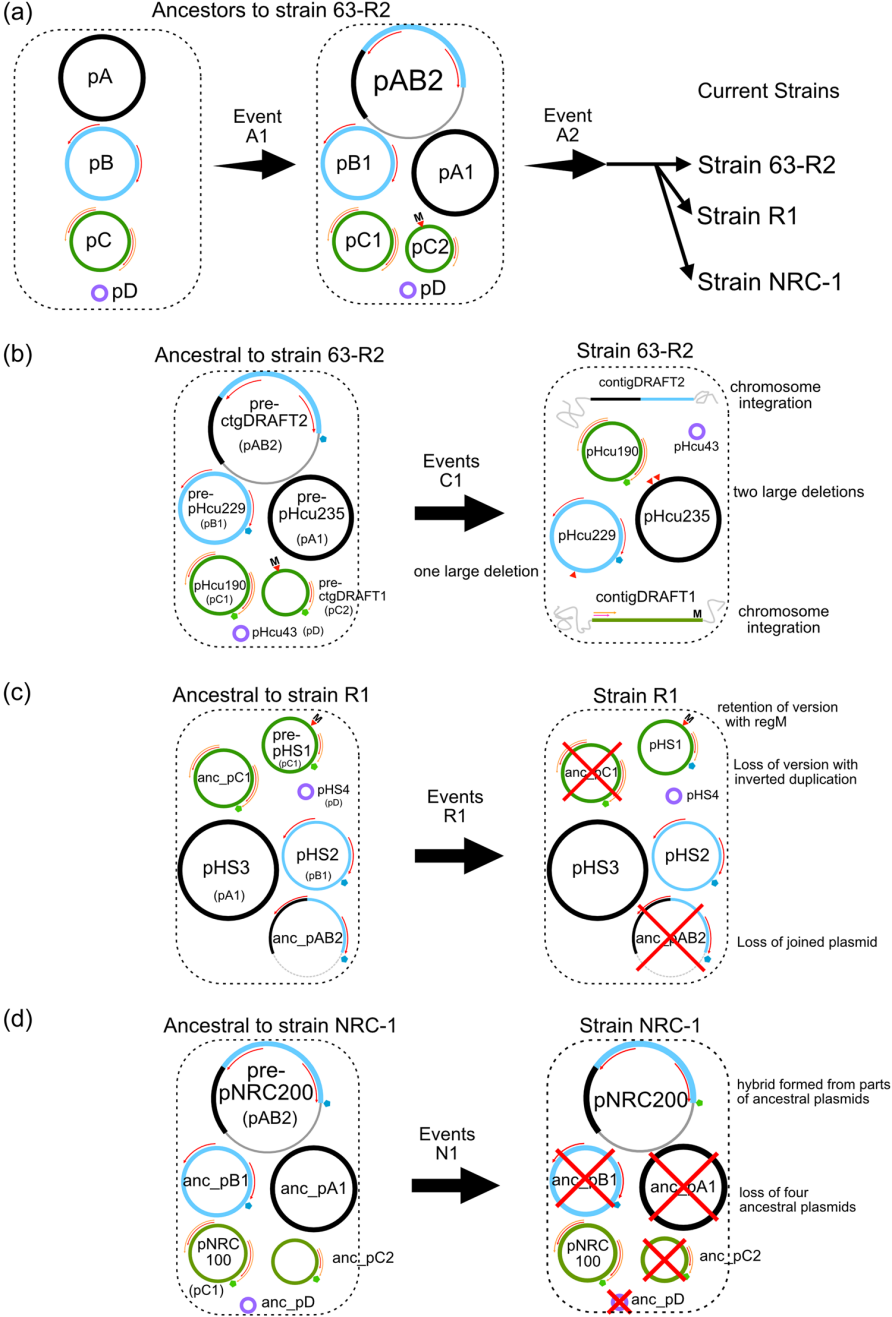
Hypothetical scenario for plasmid evolution from the common ancestor to strain 63‐R2 and further to the twin laboratory strains NRC‐1 and R1. (a, left) We hypothesize that the ancestor contained four plasmids (pA to pD) which roughly corresponds to the four strain R1 plasmids pHS3 (pA), pHS2 (pB), pHS1 (pC), and pHS4 (pD). Plasmid pB carries an inverted 32 kb repeat (outward‐facing red arrows) while plasmid pC contains an extended version (40 kb) of the inverted repeat (additional outward‐facing orange arrows). (a, center): For reasons described in the text, we hypothesize that Event A1 consists of two duplications, followed by modifications. This event occurred in the ancestor of strain 63‐R2 which is the parent of the laboratory strains NRC‐1 and R1. (i) Plasmid pC has been duplicated. One version was retained (pC1) while the other (pC2) suffered a deletion‐coupled insertion (marked by a red arrowhead labeled “M” in pC2). The deletion amounts to 58 kb while the inserted sequence is less than 2 kb. The inserted sequence (region M in Supporting Information: Table S3.3 in Supplementary Text S3: https://doi.org/10.5281/zenodo.7780801, see also Figure 3c) has been previously reported as a sequence that occurs only in strain R1 and not in strain NRC‐1 (Pfeiffer et al., 2008). The 58 kb deletion covers a 16 kb sequence (region R in Supplementary Table S3.3 in Supplementary Text S3) and the reverse copy of the 40 kb inverted duplication (InvDupCoreRev+InvDupExtraRev, Supplementary Table S3.3 in Supplementary Text S3). (ii) Plasmids pA and pB were retained in their original form (pA1, pB1) but also parts of these plasmids were duplicated and concatenated (pAB2). This concatenation joined region N (Supplementary Table S3.4 in Supplementary Text S3) and region T (Supplementary Table S3.4 in Supplementary Text S3: https://doi.org/10.5281/zenodo.7780801). (a, right) Event A2 indicates that this ancestor may have further evolved during passaging of strain 63‐R2 until samples were taken that were propagated into strains NRC‐1 and R1. It is possible that further passaging of strain 63‐R2 occurred after taking those samples, which may have resulted in additional modifications of its plasmids. (b) This panel shows the hypothesized events leading to the four plasmids of strain 63‐R2. (b, left) The plasmids hypothesized to occur in the immediate ancestor are drawn (see panel a center) but labeled to reflect the plasmids from strain 63‐R2, with labels from panel a center being provided in parenthesis. Two alternative and unrelated sequences enclosed in the same sequence context are indicated by colored pentamers. The green pentamer (in plasmids derived from pC) refers to a 4.5 kb sequence, and the blue pentamer (in plasmids derived from pB) refers to a 19.3 kb sequence. (b, right) This panel shows the four episomal plasmids of strain 63‐R2 and the two plasmid integrations into the chromosome. Two of the four episomal plasmids have been reported (DasSarma et al., 2022), pHcu235 (derived from pA1) and pHcu43 (derived from pD). Plasmid pHcu235 differs from its precursor by two closely spaced long deletions (44.2, 7.5 kb) (indicated by red triangles). One episomal plasmid has been assembled to completion and is first described in this report (pHcu190, derived from pC1). Another episomal plasmid could only be partially assembled but could be completed in silico, and is first described in this report (pHcu229, derived from pB1). Plasmid pHcu229 differs from its precursor by one long deletion (12.9 kb) (indicated by a red triangle). The alternative and unrelated sequences (colored pentamers) are retained from their precursors. The ancestral plasmid pC2 has been integrated into the chromosome (contigDRAFT1) while the free form of the plasmid has been lost. Also, parts of pC2 were lost upon chromosomal integration. The ancestral concatenated plasmid pAB2 has been integrated into the chromosome (contigDRAFT2) while the free form of the plasmid has been lost. Also, parts of pAB2 were lost upon chromosomal integration. Because plasmid integration occurred at only a single site in the chromosome, parts of pC2 and pAB2 may have been joined and further modified before their chromosomal integration. (c) This panel shows the hypothesized events leading to the four plasmids of strain R1. Plasmid pHS3 corresponds to pA1, plasmid pHS2 to pB1, and plasmid pHS4 to pD. Plasmid pHS1 corresponds to pC2 while the ancestral pC1 (anc_pC1) has been lost. Also, the concatenated ancestral plasmid pAB2 (anc_pAB2) has been lost in this strain. In pHS1, a 4.5 kb sequence (green pentamer) has been replaced by a 19.3 kb sequence from pHS2 (blue pentamer) so that the 4.5 kb sequence has been lost from strain R1. (d) This panel shows the hypothesized events leading to the two plasmids of strain NRC‐1. Plasmid pNRC100 corresponds to pC1 while the ancestral pC2 has been lost. Plasmid pNRC200 corresponds to the concatenated plasmid (pAB2) while the ancestral plasmids pA1 (anc_pA1) and pB1 (anc_pB1) have been lost. The ancestral plasmid pD (anc_pD) has also been lost. In pNRC200, a 19.3 kb sequence (blue pentamer) has been replaced by a 4.5 kb sequence (green pentamer) from pNRC100 so that the 19.3 kb sequence has been lost from strain NRC‐1.

This suggests the following hypothetical scenario for plasmid evolution from the common ancestor to 63‐R2, the parent of the twin laboratory strains NRC‐1 and R1 (Figure 4). The precursor of pHS1 and pNRC100 was duplicated in the common ancestor, one copy being converted from the pNRC100 architecture to the pHS1 architecture by deletion‐coupled insertion. Also, the precursor of pNRC200, pHcu235, and pHcu229 was duplicated in the common ancestor. Strain 63‐R2 has retained both versions. Having already partially eliminated the version with the pHS1 architecture, that version integrated into the chromosome but has then been largely but not yet completely lost from strain 63‐R2. Strain NRC‐1 received both versions but eliminated that with the pHS1 architecture. Strain R1 also received both versions but subsequently lost the version with the pNRC100 architecture. Corresponding events of duplication, partial loss in strain 63‐R2 with chromosomal integration, and complete strain‐specific deletion of the plasmid with one of the architectures have also shaped pNRC200 in strain NRC‐1 and plasmids pHS2 and pHS3 in strain R1.

Strain R1 was obtained in the Stoeckenius lab, working with “*Hbt. halobium*” (Stoeckenius & Kunau, 1968; Stoeckenius & Rowen, 1967). The Lochhead strain obtained from a buffalo hide was assigned the species epithet “*cutirubrum*,” while the epithet “*salinarum*” that had been coined by Harrison and Kennedy in 1922 (Harrison & Kennedy, 1922) was assigned to the strain obtained from a cow hide (Lochhead, 1934). TYGS analysis confirms that all these strains belong to the same species, supporting the conclusion by Ventosa and Oren (1996) that *Hbt. salinarum*, *Hbt. cutirubrum*, and *Hbt. halobium* are the same.

## AUTHOR CONTRIBUTIONS

**Friedhelm Pfeiffer**: Conceptualization (lead); formal analysis (equal); investigation (equal); project administration (lead); validation (equal); writing—original draft (equal); writing—review and editing (equal). **Mike Dyall‐Smith**: Formal analysis (equal); investigation (equal); visualization (lead); writing—original draft (equal); writing—review and editing (equal).

## CONFLICT OF INTEREST STATEMENT

None declared.

## ETHICS STATEMENT

None required.

## Figures and Tables

**Figure A2:**
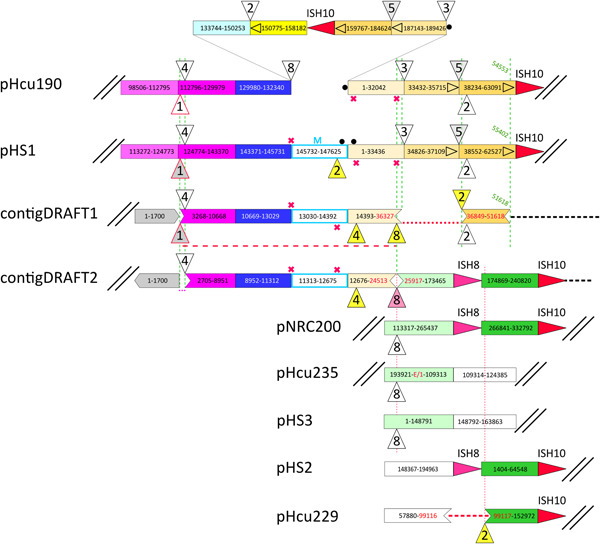
Comparative maps of the plasmids integrated into the chromosome of strain 63‐R2 with related episomal plasmids from strains 63‐R2, NRC‐1, and R1. Episomal plasmids are drawn only partially (indicated by double forward slashes at termini). Complete representations of the episomal plasmids are given in Figure A1. Most graphical elements correspond to those of Figure A1, including colored rectangles for shared regions (not drawn to scale; coordinates are given); numbered triangles for transposons; black dots indicate the first/last base of a plasmid; and arrows show the orientation of a region that is part of an inverted duplication. For graphical elements that are not described in this legend, see the legend for Figure A1. Coordinates for pHcu229 refer to the assembled version (170,458 bp) and not to the version completed in silico (229,124 bp). MGEs are frequent at region junctions or are associated with an incomplete end of a region. Most MGEs are transposons (indicated by triangles with a black outline and a number indicating the transposon type). One of the MGEs is a MITE (MITEHsal1, red outline). The targeting of an MGE by another MGE is indicated by a gray background color. Some copies of MGEs occur only in a subset of the regions. Their presence is indicated by a triangle with a yellow background, and their absence by a little cross in red. The MGE with red background color is an ISH8 which is a shared copy with respect to the region on the right and an extra copy with respect to the region on the left. Several of the shared regions are incomplete at one or both ends. A complete end of a region is indicated by a straight line with the position shown in black font. A jagged end is used to illustrate an incomplete end and the position is shown in red font (except at the beginning of contigDRAFT1 and contigDRAFT2). MGEs that are associated with incomplete region ends are shown. Dashed vertical helper lines (green or red) are drawn to facilitate the correlation between these graphical elements. Positions corresponding to the last base of contigDRAFT1 (pos 51,618) are indicated for pHS1 and pHu190. Both integrated plasmids are complete at their beginning (integration site) but partial at the other end because inter‐plasmid duplications exceed the length of PacBio reads (indicated by the black broken line). The integrated plasmids begin with chromosomal sequence (restricted to 1700 bp; arrows in gray). Arrows indicate that integration occurred in both orientations (for details, see the descriptions in Supplementary File S3: https://doi.org/10.5281/zenodo.7780801). Plasmids integrated into the forward orientation (as exemplified by contigDRAFT1) have an additional sequence of 1717 bp and begin with a targeted MITEHsal1 (triangle having a red outline, a gray background, and labeled 1). MITEHsal1 is targeted in the same way in pHS1 but not in pHcu190. There is an ISH4 transposon nearby (triangle with black outline, labeled 4), and plasmids integrated into the reverse orientation start at this ISH4. From the start of the ISH4 to the divergence point (the end of an ISH8B element), contigDRAFT1 and contigDRAFT2 are duplicated over 24,217 bp. Beyond the ISH8B element, contigDRAFT1 and contigDRAFT2 differ completely. The association of the contigDRAFT1 extension with forward integration and the contigDRAFT2 extension with reverse integration is arbitrary as PacBio reads were also found that resemble the opposite association. The 24.2 kb duplication between contigDRAFT1 and contigDRAFT2 is very closely related to plasmids pHS1 from strain R1, pHcu190 from strain 63‐R2, and pNRC100 from strain NRC‐1 (not drawn). The shared sequence begins upstream of the point where pHS1 diverges from pHcu190 and pNRC100. Plasmid pHS1 contains regionM (see Supplementary File S3, section S3.16, and Supplementary Table S3.4 at https://doi.org/10.5281/zenodo.7780801), a short region that is absent from the episomal plasmids of strains 63‐R2 and NRC‐1. These two plasmids contain a long (58 kb) sequence instead. Thus, the presence of regionM on the integrated plasmids is a notable evolutionary marker (see Text). While regionM in pHS1 carries a copy of ISH2 (indicated by a triangle with yellow background), this element is absent from contigDRAFT1 and contigDRAFT2 (indicated by little crosses in red). The 24.2 kb duplication traverses the point of ring opening of pHS1 (black dots to indicate plasmid termini) and from then onwards again also resembles pHcu190 and pNRC100 from their start. There are extra ISH4 and ISH8 elements in contigDRAFT1 and contigDRAFT2 which are not present in any of the episomal plasmids (indicated by triangles with yellow background). The ISH8 belongs to the ISH8B variant and marks the end of the duplication between contigDRAFT1 and contigDRAFT2. Beyond the ISH8B element, contigDRAFT1 corresponds to pHS1 in a region where pHS1 is near‐identical to pHcu190 and pNRC100. ContigDRAFT1 suffered a 21.2 kb deletion (loosely dashed red line) when compared to the episomal plasmids. Beyond the ISH8B element, contigDRAFT2 corresponds most closely to pNRC200. The ISH8B at this junction (triangle with red background labeled 8) is an extra copy with respect to the upstream region but a shared copy with respect to the downstream region. The region which is closely related between contigDRAFT2 and pNRC200 occurs as independent regions on distinct episomal plasmids in strains 63‐R2 (pHcu235; pHcu229) and R1 (pHS3; pHS2). Many MGEs differ between the integrated contigDRAFT2 and the episomal plasmids (see section S3.15 and Supplementary Tables S3.1 and S3.2 in Supplementary File S3, deposited at Zenodo: https://doi.org/10.5281/zenodo.7780801).

## Data Availability

Sequence data have been deposited at Zenodo: https://doi.org/10.5281/zenodo.7288901. This includes the sequences of the newly assembled plasmids pHcu190 and pHcu229 (in the assembled and the in silico completed version); the sequence of contigDRAFT1 and contigDRAFT2; the core sequences and total sequences; all sequences with tagging of the mobile genetic elements. Supplementary material has been deposited at Zenodo: https://doi.org/10.5281/zenodo.7780801. This includes Supplementary Methods, Supplementary Texts S1–S3, and Supplementary Table S4.

## 1

See Figures A1 and A2, Tables A1, A2, A3, A4, A5, A6, A7.

**Table A1:** Strain‐specific MGEs which were eliminated upon generation of the core chromosome sequences of strains 63‐R2, NRC‐1, and R1.

Serial	Category	Length (bp)	Region (63‐R2)	Region (NRC‐1)	Region (R1)	MGE type	Target sequence duplication (TSD) (bp)	Comment
1	CA	1403	8546–9948	‐	‐	ISH3B	5	
2	CA	842	10,071–10,912	‐	‐	ISH2 + ISH8A (partial)	10	Special case A
3	CA	1012	‐		35,961–36,972	ISH4	8	
4	CA	1413	‐	‐	56,795–58,207	ISH8B	10	Integration site corresponds to core genome positions 55,782 in strains 63‐R2 and R1 and 55,781 in strain NRC‐1
5	CA	531	58,039–58,569	‐	‐	ISH2	10	Integration site corresponds to core genome positions 55,793 in strains 63‐R2 and R1 and 55,792 in strain NRC‐1
6	CA	1413	‐	56,171–57,583	‐	ISH8B	10	Integration site corresponds to core genome positions 56,174 in strains 63‐R2 and R1 and 56,173 in strain NRC‐1
7	CA	1130	61,642–62,771	‐	‐	ISH1	8	
8	CA	1413	90,788–92,200	‐	89,307–90,719	ISH8B	10	
9	CA	1403	‐	95,825–97,227	‐	ISH3B	5	
10	CA	1394	‐	99,327–100,720	‐	ISH3C	5	
11	CA	531	103,081–103,611	‐	‐	ISH2	10	
12	CA	531	119,673–120,203	‐	117,661–118,191	ISH2	10	
13	CA	531	‐	176,934–177,464	‐	ISH2	10	Special case B
14	CB	1413	267,225–268,637	265,581–266,993	255,729–257,141	ISH8B	10	
15	CA	1130	‐	‐	289,699–290,828	ISH1	8	
16	CA	1456	533,027–534,482	‐	‐	ISH6	8	
17	CA	1012	‐	697,026–698,037	‐	ISH4	8	
18	CA	1403	753,135–754,537	‐	‐	ISH8E	None	Special case C
19	CA	1394	‐	‐	741,710–743,103	ISH3C	5	
20	CA	1394	‐	‐	744,664–746,057	ISH3C	5	
21	CA	531	762,082–762,612	‐	‐	ISH2	10	Integration site corresponds to core genome position 752,839 in strain 63‐R2, 752,882 in strain NRC‐1 and 743,400 in strain R1
22	CA	531	‐	759,508–760,038	‐	ISH2	10	Integration site corresponds to core genome position 753,711 in strain 63‐R2, 753,754 in strain NRC‐1 and 744,272 in strain R1
23	CA	1394	765,891–767,284	‐	‐	ISH3C	5	Integration site corresponds to core genome position 756,117 in strain 63‐R2, 756,160 in strain NRC‐1 and 746,678 in strain R1
24	CA	531	‐	771,563–772,093	‐	ISH2	10	Integration site corresponds to core genome position 765,235 in strain 63‐R2, 765,278 in strain NRC‐1 and 755,796 in strain R1
25	CA	1130	990,664–991,793	‐	978,358–979,487	ISH1		
26	CA	1074	‐	1,184,712–1,185,785	‐	ISH11	6	
27	CA	1394	‐	1,186,471–1,187,864	‐	ISH3C	5	
28	CA	532	‐	‐	1,220,109–1,220,640	ISH2	11	Integration site corresponds to core genome position 1,220,155 in strain 63‐R2, 1,220,173 in strain NRC‐1 and 1,210,691 in strain R1
29	CA	531	‐	1,230,337–1,230,867	‐	ISH2	10	Integration site corresponds to core genome position 1,221,035 in strain 63‐R2, 1,221,053 in strain NRC‐1 and 1,211,571 in strain R1
30	CA	1413	‐	1,231,045–1,232,457	‐	ISH8E	10	
31	CA	1413	‐	1,608,077–1,609,489	‐	ISH8B	10	
32	CB	1853	1,987,893–1,989,745	1,987,839–1,989,691	1,975,957–1,977,809	ISH34	‐	An IS605‐type element; MGEs of this type never generate a TSD
33	CA	1394	‐	2,004,215–2,005,608	‐	ISH3C	5	

*Note*: Mobile genetic elements (MGEs) of category CA were deleted when comparing these three strains to each other. MGEs of category CB are common to these three strains but are strain‐specific when compared to strain 91‐R6. In each case, the length of the removed sequence (MGE + TSD) is given, and the position in the affected strain. A dash is given for nonaffected strains. Coordinates refer to the original chromosome sequence. The type of strain‐specific MGE is indicated. Nearly all strain‐specific MGEs were found to be associated with a TSD, the length of which is specified. If strain‐specific MGEs were very closely spaced, their relative positioning is specified in the comment column by their integration positions in the three core genomes. Special cases requiring an extended description are labeled as “special case” in the comment (see hereafter). Special case A: A complete ISH2 and a partial ISH8A element (terminal 311 bp) were integrated as a cassette, which is concluded from the fact that the two elements are directly adjacent and are bounded by one common 10 bp target duplication. It should be noted that ISH2 is a MITE, which does not carry a transposase gene, has termini related to those of ISH8 and is mobilized in trans by the ISH8 transposase. Special case B: This ISH2 is present in the integrative element insert of strain NRC‐1 but absent from the corresponding element in strain 63‐R2. The integrative element is completely absent from strain R1 (see Figure 2). Special case C: not associated with a TSD; the sequence TC‐GT‐GT‐AT‐GT‐CT (strains NRC‐1 and R1) is replaced by TC‐GT‐GT‐[ISH8E]‐GT‐CT (strain 63‐R2).

**Table A2:** Strain‐specific MGEs which were eliminated upon generation of the core chromosome sequence of strain 91‐R6.

Serial	Category	Length (bp)	Region (91‐R6)	MGE type	Target sequence duplication (TSD) (bp)
1	CB	1394	1,027,440–1,028,833	ISHsal1	5
2	CB	1657	1,203,639–1,205,295	ISNpe8	7
3	CB	413	1,408,020–1,408,432	MITEHsal2	8
4	CB	411	1,455,597–1,456,007	MITEHsal2	6
5	CB	1592	1,593,564–1,595,155	ISH10	8

*Note*: Mobile genetic elements (MGEs) deleted from the chromosome of strain 91‐R6 upon generation of the core sequence. These MGEs are strain‐specific when compared to the chromosomes of the three strains 63‐R2, NRC‐1, and R1. See Table A1 for additional explanations.

**Table A3:** Strain‐specific mobile genetic elements (MGEs) which were eliminated upon generation of the core plasmid sequences pHcu235 and pHS3.

Serial	Length (bp)	Region (pHcu235)	Region (pHS3)	MGE type	Target sequence duplication (TSD) (bp)
1	1394	206,223–207,616	‐	ISH3C	5
2	1403	220,507–221,909	‐	ISH3B	5
3	531	233,778–234,308	‐	ISH2	10
4	1403	‐	140,883–142,285	ISH3B	5
5	1389	175,248–176,641	‐	ISH3D	5

*Note*: See Table A1 for explanations.

**Table A4:** Strain‐specific mobile genetic elements (MGEs) which were eliminated upon generation of the core plasmid sequences pHcu43 and pHS4.

Serial	Length (bp)	Region (pHcu43)	Region (pHS4)	MGE type	Target sequence duplication (TSD) (bp)
1	531	39,231–39,761	‐	ISH2	10
2	1394	7287–8680	‐	ISH3D	5
3	1413	12,184–13,596	‐	ISH8B	10
4	1413	‐	30,920–32,332	ISH8B	10

*Note*: See Table A1 for explanations.

**Table A5:** Strain‐specific MGEs which were eliminated upon generation of the core plasmid sequences pHcu190, pNRC100, and pHS1.

Serial	Length (bp)	Region (pHcu190)	Region (pNRC100)	Region (pHS1)	MGE type	Target sequence duplication (TSD) (bp)	Comment
1	1394	‐	‐	22,454–23,847	ISH3C	5	
2	1076	36,765–37,840	36,765–37,840	‐	ISH11	8	
3	531	‐	‐	40,645–41,175	ISH2	10	
4	1413	58,502–59,914	58,502–59,914	‐	ISH8B	10	
5	531	70,690–71,220	70,690–71,220	‐	ISH2	10	
6	1403	81,981–83,383	81,981–83,383	‐	ISH3B	5	
7	1413	‐	‐	104,639–106,051	ISH8B	10	
8	1013	‐	‐	106,149–107,161	ISH4	9	
9	1394	101,441–102,834	101,441–102,834	‐	ISH3D	5	
10	1394	105,570–106,963	105,570–106,963	‐	ISH3C	5	
11	1413	‐	‐	134,541–135,953	ISH8B	10	
12	531	‐	153,539–154,069	‐	ISH2	10	Region absent from pHS1

*Note*: Several mobile genetic elements (MGEs) are shared between strains 63‐R2 and NRC‐1 but absent from strain R1. In the forward copy of the long (40 kb) inverted duplication, they are considered strain‐specific. Their counterpart in the reverse copy of the inverted duplication is not considered to be strain‐specific because the inverted copy is absent from strain R1 and thus only strains 63‐R2 and NRC‐1 are compared, both containing this MGE. See Table A1 for additional explanations.

**Table A6:** Strain‐specific mobile genetic elements (MGEs) which were eliminated upon generation of the core plasmid sequences pHcu229, pNRC200, and pHS2.

Serial	Length (bp)	Region (pHcu229)	Region (pNRC200)	Region (pHS2)	MGE type	Target sequence duplication (TSD) (bp)	Comment
1	1413	‐	58,502–59,914	‐	ISH8B	10	
2	531	‐	70,690–71,220	‐	ISH2	10	
3	531	27,835–28,365	‐	‐	ISH2	10	
4	1394	53,282–54,675	‐	‐	ISH3D	5	ISH3D in pHcu229 and ISH3B in pNRC200 are integrated into an identical sequence context
5	1403	‐	81,981–83,383	‐	ISH3B	5	ISH3B in pNRC200 and ISH3D in pHcu229 are integrated into an identical sequence context
6	531	60,732–61,262	‐	‐	ISH2	10	
7	1077	81,558–82,634	‐	‐	ISH11	9	
8	531	‐	308,738–309,268	‐	ISH2	10	
9	531	‐	‐	61,277–61,807	ISH2	10	
10	1394	156,244–157,637	‐	‐	ISH3D	5	

*Note*: For pHcu229, the analysis is restricted to its assembled part and coordinates refer to the assembled version, not to the in silico completed version. See Table A1 for additional explanations.

**Table A7:** The extent of the AT‐rich regions in strains 63‐R2, NRC‐1, and R1 and the equivalently positioned replacement region in strain 91‐R6.

Strain	Position	Length (bp)	G + C (%)	Protein range
91‐R6	216,886–264,382	47,497	56.4	HBSAL_01115 to HBSAL_01355
63‐R2	12,851–74,116	61,266	56.1	LJ422_00060 to LJ422_00390
NRC‐1	10,606–71,619	61,014	56.1	VNG_0011C to VNG_0080H
R1	10,606–72,635	62,030	56.2	OE_1018F to OE_1136F

*Note*: For defining the left and right boundary of the replacement region of the two Lochhead strains, which corresponds to the AT‐rich region in strains 63‐R2, NRC‐1, and R1, see Supplementary text S2, deposited at Zenodo: https://doi.org/10.5281/zenodo.7780801, section S2.4. Coordinates refer to the original genome sequence (for accessions, see Table 1).
